# The use of silicon-based tethers for the Pauson-Khand reaction

**DOI:** 10.1186/1860-5397-3-21

**Published:** 2007-07-06

**Authors:** Adrian P Dobbs, Ian J Miller, Saša Martinović

**Affiliations:** 1 School of Biological and Chemical Sciences, Walter Besant Building, Queen Mary University of London, Mile End Road, London E1 4NS, UK; 2 Department of Chemistry, University of Exeter, Stocker Road, Exeter, E4 4QD, UK

## Abstract

A range of silicon-based tethers and promoters have been investigated for use in the development of a silyl-tethered Pauson-Khand reaction.

## Background

Since its discovery in 1973, the Pauson-Khand (P-K) reaction has become one of the principal methods for the construction of cyclopentenones.[1–2]

Temporary tethers have long been used to convert an intermolecular reaction to an intramolecular one and thus favour reaction. Silicon is by far the most popular choice of element when considering forming a temporary tether to link two reaction components.[3] This popularity is due to several factors. First, the acyclic silicon containing chains are simple to synthesise, such as through the formation of either silyl ethers or acetals, may contain a wide range of functionalities and are stable to a range of different reaction conditions and purification techniques. Second, the silicon tethers remain inert in most reactions but they can be easily and selectively removed using fluoride containing compounds, such as tetrabutylammonium fluoride (TBAF), or by using the Tamao-Fleming oxidation procedure. In addition, the silicon may also be used simultaneously to protect functionalities during the reaction sequences. Recent examples of the use of silicon-containing tethers have centred upon the Diels-Alder reaction,[4–5] radical reactions[6] and olefin metathesis reactions.

**Scheme 1:**
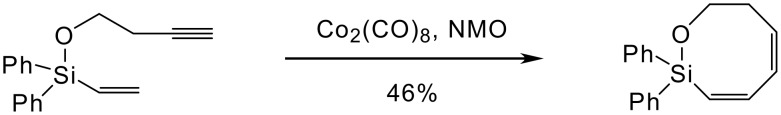
Saigo's cycloisomerisation reaction under Pauson-Khand conditions.

There have been reports of applying the temporary tethering methodology of silicon species to the P-K reaction, but with limited success. Saigo reported that the attempted P-K cyclisation of a variety of 3-sila-1,7-enynes underwent cycloisomerisation instead of the cycloaddition (Scheme 1).[7]

**Scheme 2:**
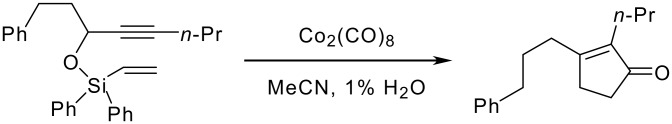
Pauson-Khand reaction and tether-cleavage in wet acetonitrile.

Saigo's work showed that this cycloisomersiation was only applicable to 3-sila-1,7-enynes and any other chain length would undergo decomposition. Pagenkopf has shown that when the P-K cyclisation is carried out in 'wet' acetonitrile the cyclisation would proceed to give the cyclopentenones (Scheme 2).[8–9] The tethering strategy was not however successful in that although cyclisation gave the correct regiochemistry, the silicon tether is cleaved from the molecule by the reaction conditions and leaves no functionality for further synthetic modifications.

**Scheme 3:**
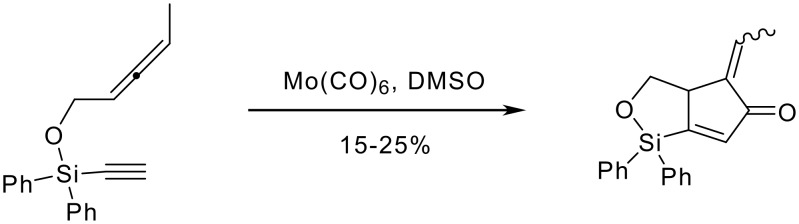
Silyl-tethered allenic Pauson-Khand reaction reported by Brummond.

There are only two examples of silicon tethers being successfully applied to a Pauson-Khand type reaction. Brummond researched a large variety of potential systems but found that silicon tethers did not seem to be compatible with the Pauson-Khand reaction. Fortunately her research discovered that by combining the silicon tether with the allenic Pauson-Khand reaction mediated by molybdenum hexacarbonyl the corresponding bicyclic cyclopentenone could be formed although with poor yields (Scheme 3).[10–11]

**Scheme 4:**
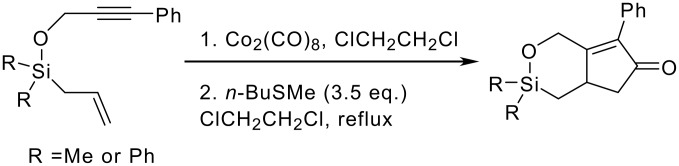
Intramolecular Pauson-Khand reaction of allyldimethyl- and allyldiphenylsilyl propargyl ethers reported by Porter.

Finally, in a recent report, Porter has described the intramolecular Pauson-Khand reaction of allyldimethyl- and allyldiphenylsilyl propargyl ethers promoted by dicobalt octacarbonyl and *n*-butyl methyl sulphide as a promoter to give the bicycles in modest to good yields (Scheme 4).[12]

It can be seen that although the silicon methodology has been applied to the P-K reaction no group has been able to combine the synthetic diversity of silicon tethers with the synthetic benefits of the dicobalt octacarbonyl mediated cyclisation of alkynes and alkenes.

## Results and Discussion

We decided to carry out a thorough investigation of the potential for development of a silicon-tethered Pauson-Khand reaction, using three different types of tether.

### i) Silyl ether tethers

Both vinyldimethylchlorosilane and vinyldiphenylchlorosilane are commercially available and were chosen as the initial starting materials for this part of the study. A range of silyl ethers were formed, which were then subjected to the 'standard' Pauson-Khand reaction conditions of dicobalt octacarbonyl and *N*-methylmorpholine *N*-oxide.

**Scheme 5:**
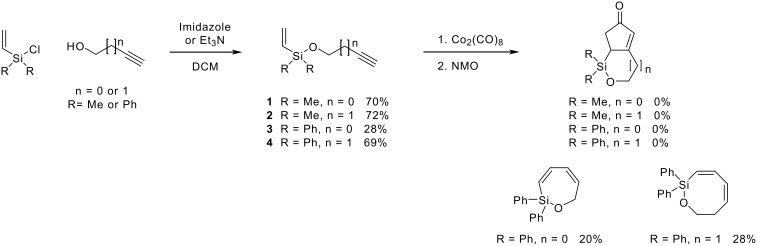
Synthesis and attempted Pauson-Khand reactions of vinyldimethylsilyl- and vinyldiphenylsilyl ethers.

Although the silyl ethers were formed in good yields, no Pauson-Khand adducts were obtained, only the cycloisomerisation products predicted by Saigo.[7] Repeating the reactions under 1 atm pressure of carbon monoxide also gave only the isomerisation products, albeit in higher yields and more rapidly. In every example, the main product, accounting for the bulk of the mass balance, were silanols derived from decomposed corresponding silyl ethers.

**Figure 1:**
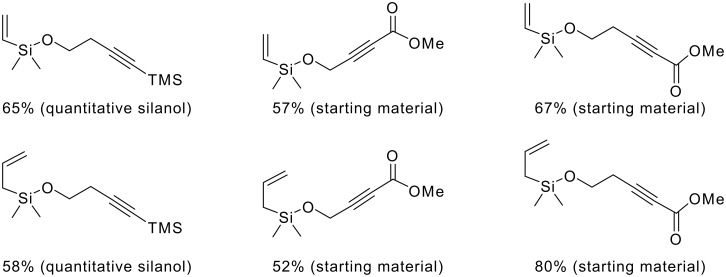
Functionalised acetylenes prepared and used in silyl ether-tethered Pauson-Khand reactions. Yields correspond to yield of the silyl ether formed, figures in brackets from the Pauson-Khand reaction.

The P-K reaction is known to be affected by steric and electronic effects within the cyclisation precursors. Therefore we prepared the following dimethyl vinyl- and allyl-silyl ethers with various groups attached to the terminus of the alkyne (Figure 1).[13–15] Once again, all the ethers were formed in good yields, but unfortunately either the silanol or starting materials were recovered in each case, as indicated, from the Pauson-Khand reaction.

**Figure 2:**
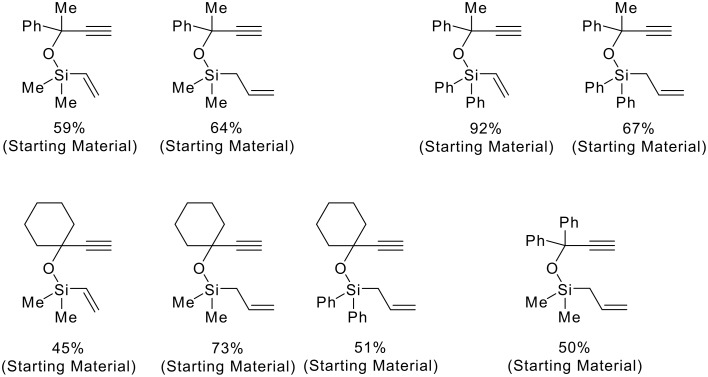
Chain-functionalised acetylenes prepared and used in silyl ether-tethered Pauson-Khand reactions. Yields correspond to yield of the silyl ether formed, figures in brackets the product recovered from the Pauson-Khand reaction.

Fearing that the lack of cyclisation may have been due to the two arms of the tether simply not coming together, substituents were introduced to the tether chains, in an attempt to produce a Thorpe-Ingold-type effect and force the two ends of the chain together (Figure 2).

There now exist a plethora both of alternative metal carbonyls and promoters for the Pauson Khand reaction. Using each of the compounds **1–4**, we first tested five alternative promoters to NMO. These were cyclohexylamine[16]; 1,4-dioxane/2M ammonium hydroxide[17]; trimethylamine *N*-oxide, 4 molecular sieves[18]; *n*-butylmethylsulfide[19] and microwave irradiation[20]. As previously, the dicobalt octacarbonyl complexes of each compound were first prepared and characterised, prior to addition of the promoter. Unfortunately, none of the promoters gave any of the desired products but simply de-complexed starting materials were recovered in each case.

**Figure 3:**
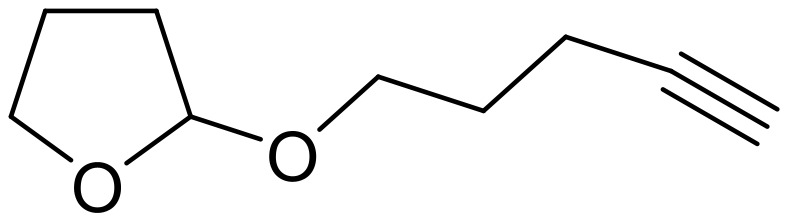
Possible structure of THF-oxidation/insertion product.

Alternative metal carbonyls were also investigated, with compounds **1–4** being reacted with each of molybdenum hexacarbonyl/DMSO[21]; tungsten pentacarbonyl/THF[22]; chromium hexacarbonyl and rhodium cycloooctadiene chloride dimer/pentafluorobenzaldehyde[23–24]. None of the promoters gave any Pauson-Khand adducts, although an interesting THF-insertion adduct was obtained from the reaction of allyldimethylpent-4-ynyloxysilane with tungsten pentacarbonyl, possibly formed *via* oxidation of THF to give an oxonium ion followed by addition of the alcohol cleaved from the silyl ether (Figure 3).

**Scheme 6:**
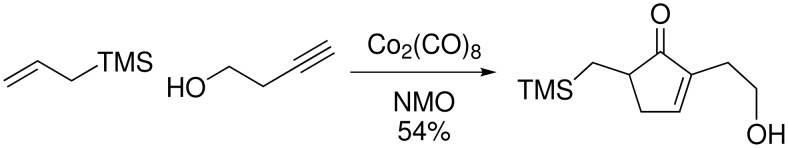
Model Pauson-Khand reaction of allyltrimethylsilane.

In order to investigate if the presence of the silicon linker was preventing the Pauson-Khand reaction occurring, a test reaction between allyltrimethylsilane and 3-butyn-1-ol was performed using dicobalt octacarbonyl and NMO (Scheme 6). A mixture of cyclopentenone regioisomeric isomers were obtained, with the principal regioisomer being the one shown in Scheme 6.

One possible explanation for the failure of all these reactions was that the 'arms' of the silyl ethers were too far apart for cyclisation to occur. We had already attempted to overcome this potential hurdle by the introduction of functionality within the side chains. Work by Denmark[25] on tethered nitrone cycloadditions has shown that for cycloaddition reactions to occur, the non-reactive substituents around the silicon centre must be more bulky than the Me or Ph groups employed in these studies. Denmark's work demonstrated that the angles at the silicon centre between the two 'arms' of the silyl ether can be up to 180°. This large angle would mean that the 'arms' would never be close enough together to undergo cycloaddition. Therefore the angle must be decreased and this can be accomplished by increasing the size of the non-reactive substituents as stated by the Thorpe-Ingold effect. Denmark stated that the substituents on silicon should either be two *iso*propyl or two *tert*-butyl groups in order to achieve reaction. Di*tert*butyl silanes were found to be impractical because vinyl- or allyldi*tert*butyl chlorosilanes will not undergo nucleophilic substitution to yield the silyl ethers due to the large steric crowding, preventing the formation of the penta coordinate intermediates. However, di*iso*propylsilanes were successful in Denmark's studies.

The preparation of di*iso*propyl silyl ethers presented a greater synthetic challenge than the previous silyl ethers. Starting from di*iso*propyldichlorosilane, a two-step, one-pot procedure was developed, initially adding the allyl arm *via* the allyl Grignard reagent, followed by a more standard silyl ether formation using an acetylenic alcohol and imidazole (without isolating the intermediate silyl chloride). (Isolation of the intermediate di*iso*propylallylsilyl chloride was impossible, since any attempt to work-up the Grignard reaction resulted in hydrolysis of the silyl chloride to the allyldi*iso*propylsilanol).

**Scheme 7:**
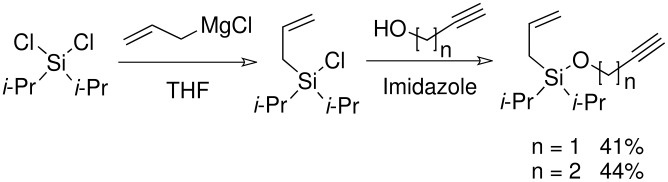
Preparation of allyldi*iso*propylsilyl ethers.

The cyclisation of these two materials was then performed using the standard Pauson-Khand reactions that had previously been successful in our model studies – dicobalt octacarbonyl and NMO.

**Scheme 8:**
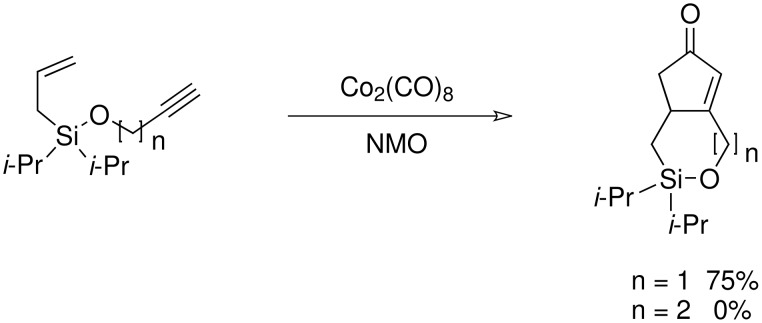
Pauson-Khand reaction of allyldi*iso*propylsilyl ethers.

Under these conditions, a very pleasing 75% yield was obtained for the fused 6,5-ring system (n = 1). This is the first example of a di*iso*propylsilyl ether-tethered Pauson-Khand reaction successfully taking place. See Supporting Information File 1 for full experimental details. Unfortunately, no reaction product was obtained for the 5,7-ring system (n = 2), although this is not quite so surprising, given the general difficulty in forming 5,7-bicyclic systems.[26]

Efforts to prepare further, more substituted allyldi*iso*propyl silyl ethers by this two-step, one-pot procedure failed to give cyclisation precursors in any appreciable yield and not sufficient for use in the Pauson-Khand reaction. The previous method had shown that the Grignard addition to a dichlorosilane had worked well but that the work-up had hydrolysed the remaining silyl chloride bond. Therefore replacing the second chlorine atom with a group that could not be hydrolysed would allow the work-up and isolation of the products after the Grignard addition had taken place. Due to the restricted number of chlorodi*iso*propylsilanes available meant that this group had to be a proton. Therefore it was decided to start this new methodology from chlorodi*iso*propylsilane.

**Scheme 9:**
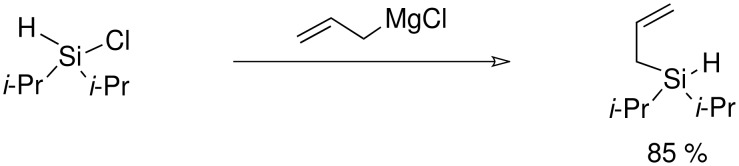
Preparation of allyldi*iso*propylsilanes.

**Scheme 10:**
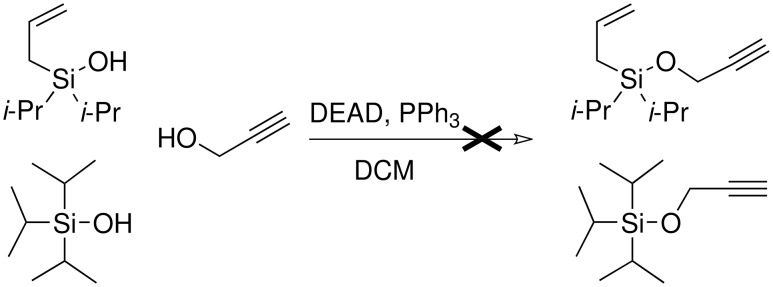
Attempted Mitsunobu reactions of di*iso*propylsilanols.

The synthesis of allyldi*iso*propylsilane proceeded easily and with high yield. Next a variety of methods were attempted for the conversion of the silicon-hydrogen bond to a silicon-chloride bond: chlorine in carbon tetrachloride; copper (II) chloride and Hunig's base; tin (IV) chloride[27] and *N*-bromosuccimide[28] were all tested but none were successful as either no reaction occurred or the alkene was halogenated as well as the conversion of the silane to the silyl chloride. Further, given that the major product from many of the methods attempted both for the formation of the silyl ethers and from the Pauson-Khand reaction were the corresponding silanols, we wondered if it would be possible to use these compounds for the preparation of our desired ethers *via* a Mitsunobu reaction. There are examples in the chemical literature in which silanols may be used analogously to alcohols in the Mitsunobu reaction.[29] Unfortunately, neither tri*iso*propylsilanol (synthesised by the hydrolysis of commercially available tri*iso*propylsilyl chloride) or di*iso*propyl(1-methylallyl)-silanol and but-2-enyldiisopropylsilanol gave any product and quantitative starting materials were recovered (Scheme 10).

Following the failure of the different methods of forming the silyl ethers it was decided to find a procedure for the direct conversion of the easily synthesised allyldi*iso*propylsilane to the silyl ethers. The first reaction used a neat mixture of the silane and alcohol with the addition of a catalytic amount of Wilkinson's catalyst.[30] A test reaction using this procedure was carried out to couple allyldi*iso*propylsilane and propargyl alcohol, but the formation of the silyl ether did not occur and the starting materials were recovered in quantitative amounts.

The second method involved dissolving the silane and alcohol in *N*-methylpyrrolidinone (NMP) followed by the addition of a catalytic amount of a 1 M solution of tetrabutylammonium fluoride (TBAF) in THF.[31] This proved to be successful and a number of different alcohols were tried and the results, together with those from the Pauson-Khand reaction are shown in Table 1.

**Table 1:** Di*iso* propylsilyl ether formation and subsequent Pauson-Khand reactions

	**Alcohol**	**Silyl Ether**	**Yield (%)**	**Pauson-Khand Adduct**	**Yield (%)**

1.	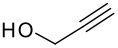	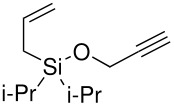	41	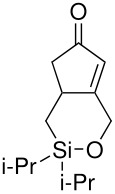	75
2.	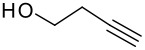	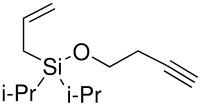	44	-	0
3.	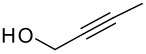	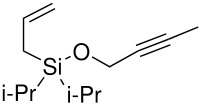	51	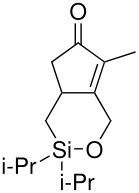	46
4.	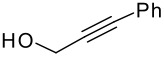	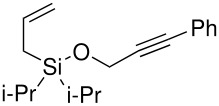	49	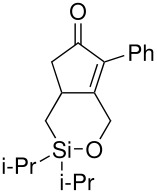	31
5.	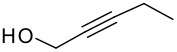	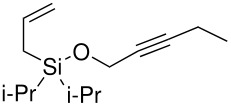	44	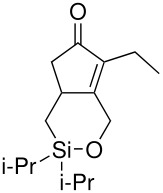	34
6.	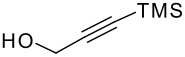	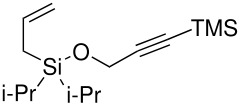	0	-	-
7.	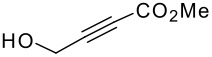	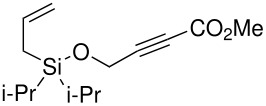	0	-	-
8.	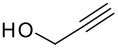	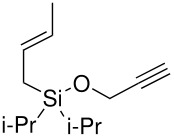	17	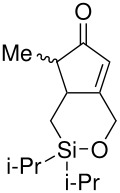	Traces
9.	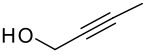	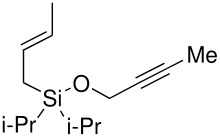	-	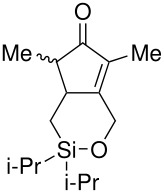	0

The application of the TBAF catalyst to the formation of silyl ethers showed mixed results. Entries 1–5 proceeded with moderate to good yields. These yields were much better than those obtained from the one-pot synthesis starting from dichlorodi*iso*propylsilane described previously. These successful reactions used the simple hydroxyl containing alkynes; when these alkynes had more functionalised substituents (entries 6 and 7) the reaction failed. In the case of entry 7 the only compound recovered from the reaction was the allyldi*iso*propylsilanols. However in the case of the TMS derivatised alkyne, the major product was the de-silylated silyl ether consistent with entry 1. The reaction proved to be very unreliable and the purity of the substrates had to be very high. Impurities, especially water, were thought to interfere with the mechanism of catalysis.

The successfully prepared silyl ethers (Table 1, entries 1–5, 8) were then used as substrates for the P-K cyclisation. The standard conditions, used previously, of one equivalent of dicobalt octacarbonyl and ten equivalents of *N*-methylmorpholine *N*-oxide (NMO) were employed. In all cases except for the longer chain (entry 2) the P-K adduct was obtained in poor to moderate yield. Only traces of product (by GCMS) were observed for entry 8, presumably due to very small scale reaction owing to the poor yield of starting material. The cyclised silyl ether tethered cyclopentenone was again synthesised proving the reaction could be repeated and was not an anomaly. The results for the other silanes did not show the product of the reaction as cyclopentenones. The only identifiable product isolated from any of the other reactions was the silanol associated with hydrolysis of the silicon-oxygen bond. It was impossible to improve on these reaction yields, despite varying the amount of NMO (1, 5 or 10 eq.) and reaction temperature (r.t., 40°C or 80°C).

Finally, it was decided to swap the alkene and alkyne substituents on the silyl ethers around. In order to achieve the formation of these new silyl ethers an alternative methodology had to be applied to both the formation of the silanes and the subsequent formation of the silyl ethers.

Simple deprotonation of the alkyne with *n*-butyllithium and reaction with chlorodi*iso*propylsilane led to the formation of the desired silanes in good yields.

**Scheme 11:**
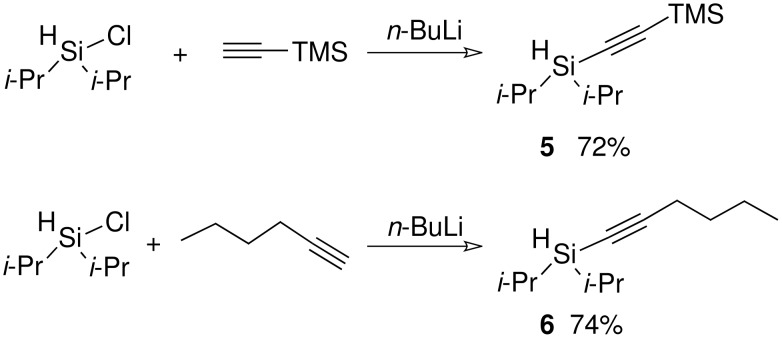
Preparation of alkynic di*iso*propylsilanes.

The formation of the silyl ethers was attempted using the TBAF catalyst procedure that had proven to be successful previously but neither compound gave the desired silyl ethers. GCMS data suggested that the TBAF catalyst had attacked the bond between the TMS group and the alkyne in the case of silane (**5**) and in the case of silane (**6**) the reaction had simply not worked, although no clear results were obtained by NMR. It has been shown that a ruthenium catalyst can cause the direct formation of silyl ethers from the silane and an alcohol. The procedure was successful for hex-1-ynyldi*iso*propylsilane.

**Scheme 12:**
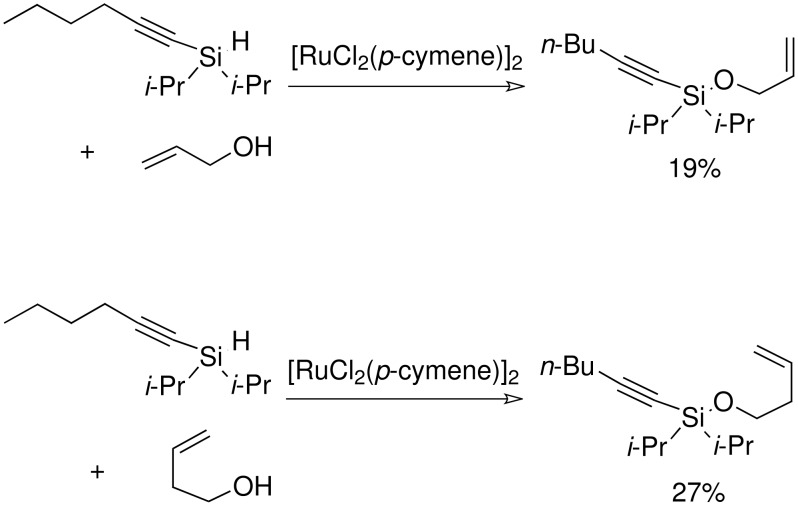
Preparation of allyldi*iso*propylsilyl ethers.

The reaction proceeded with low yields but the un-reacted silane was recovered intact at the end of the reaction. The yields for the reactions were significantly below the near quantitative yields reported in the literature but these reactions were never optimised. These two silyl ethers were subjected to the standard P-K reaction conditions. Analysis of the reaction solution showed that cyclisation had not occurred and the only compound recovered was a quantitative amount of the starting material.

### ii) Silyl acetal tethers

Although silyl ethers have been the predominant ether of choice, silyl acetals have been applied as temporary tethers in reactions.[32] Silyl acetals have, for example, been applied to any reactions to which silyl ether have been applied, such as radical, Diels Alder reaction or ring closing metathesis, albeit with varying degrees of success. The advantage of silyl acetals over silyl ethers is their greater stability to hydrolysis. The results obtained from the research into silyl ethers suggested that if, as we believe, hydrolysis was the major problem, this potentially could be overcome using silyl acetals.

First, we attempted to form the required mixed silyl acetal from propargyl and allyl alcohols using diphenyldichlorosilane and imidazole as the base and allowing the reaction to proceed to equilibrium, hopefully allowing for the optimum yield of the mixed acetal.

**Scheme 13:**
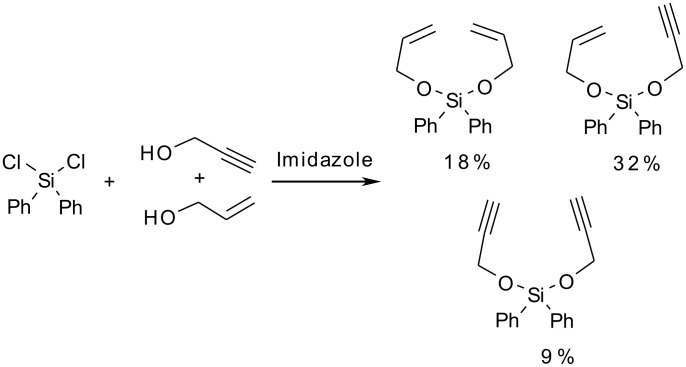
Preparation of acetals from dichlorodiphenylsilane.

The result shows that the desired mixed acetal is the major product of the reaction as expected. However, given the similarity in the three products, purification of the acetals by column chromatography proved to be particularly difficult and complete purification could not be achieved (yields given are of pure products obtained; the remaining mass of the reaction could not be completely purified and remained as two mixtures of the acetals).

**Scheme 14:**
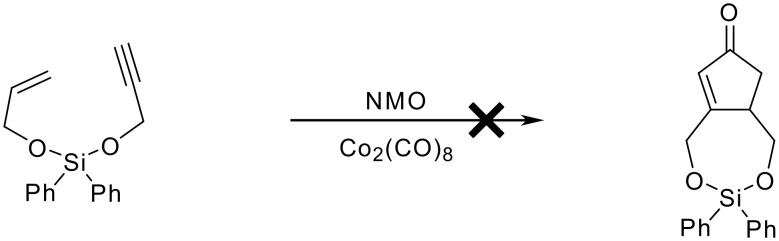
Attempted Pauson-Khand reaction of allylpropargyldiphenylsilyl acetal.

The cyclisation of the purified mixed acetal was attempted using the standard reactions conditions which had been employed for the successful silyl ether cyclisation reactions (Scheme 14).

The cyclisation failed to yield any of the bicyclic cyclopentenone predicted. This result is consistent with the literature evidence and our previous results that the 5,7 systems are known not to be synthesised through the cobalt mediated methodology. The unsymmetrical acetal was recovered in near quantitative yield with no trace of any products of decomposition or hydrolysis. Therefore silyl acetal did not undergo hydrolysis thus proving that the silyl acetal is more stable to the P-K reaction conditions than the silyl ethers.

In order to try to achieve cyclisation, the length of each 'arm' of the silyl acetal was increased by 1 carbon unit. It was hoped that the increase in chain lengths would allow the larger ring system (5,9) to be synthesised. This was accomplished by employing 3-butyn-1-ol and 3-buten-1-ol in place of propargyl and allyl alcohol respectively. The same experimental procedure was used and the three acetals were formed in roughly the same ratio as the previous attempt. Unfortunately, on this occasion, purification by chromatography was completely unable to isolate any of the pure mixed acetal. It was found that the increased chain length had decreased the differences in polarity to such a degree that separation by chromatography was impossible. Purification by distillation proved to be similarly impossible.

**Scheme 15:**
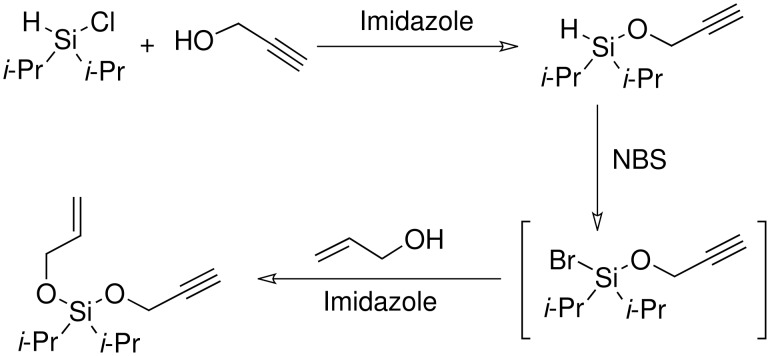
Proposed di*iso*propylsilyl acetal formation.

Following the work of Denmark and our moderate success in the silyl ether series, it was decided to attempt to use di*iso*propylsilanes as the base for the acetals. Secondly it was decided to find a methodology that allowed for the formation of only the mixed acetal. In order to achieve this it was thought to form each of the 'arms' of the acetal in separate synthetic steps (Scheme 15).

**Scheme 16:**
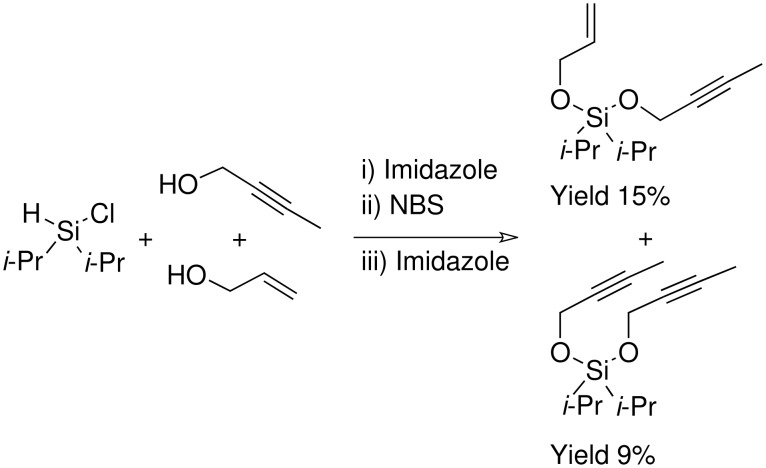
Attempted allylpropargyldi*iso*propylsilyl acetal formation.

The first stage of the reaction was silyl ether formation. The alkyne 'arm' of the acetal was introduced first, as it was feared that during the next, halogenation step, halogenation of the alkene might occur, if present. This proved to be a successful approach and it was not necessary to purify the first reaction, but simply continue to perform the silyl acetal. The first reaction was attempted using 2-butyn-1-ol and allyl alcohol (Scheme 16).

The result demonstrates that the synthesis of the mixed acetal is successful. However the symmetrical di-alkyne acetal is also formed. This is due to the formation first of the silyl acetal 'arm' not going to completion. Thus after bromination there is still some 2-butyn-1-ol remaining in solution and this reacts with the bromosilane. The low yields could be improved by optimizing the reaction conditions and finding a better way to add the second arm to the acetal, such as a catalytic method, avoiding the need for the bromosilane intermediate. This reaction was repeated using 3-butyn-1-ol to yield the isomeric, terminal alkyne product. However only the symmetrical, di-alkyne acetal was isolated from the reaction mixture and the yield was poor (14%). The poor yield was again due to the bromosilane hydrolysing under the reaction conditions.

**Scheme 17:**
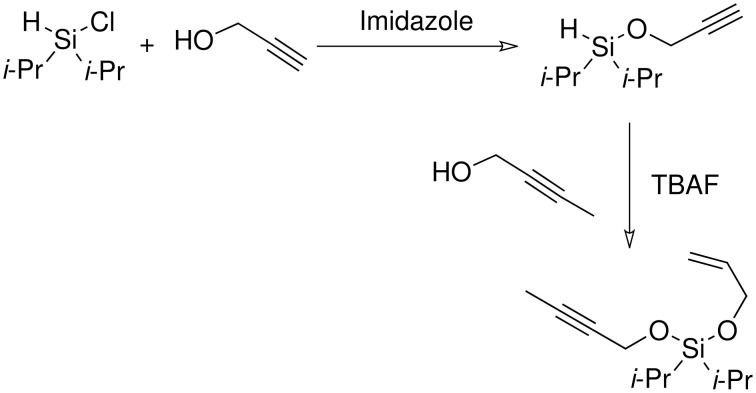
Attempted allylpropargyldi*iso*propylsilyl acetal formation.

The final methodology for synthesising the silyl acetals hoped to combine the silyl ether formation utilised in the NBS procedure with the TBAF methodology which had been so successful for the silyl ethers (Scheme 17).

The formation of the alkene 'arm' of the acetal proceeded well using the methodology outlined above. The TBAF catalysed addition of the alkyne 'arm' however did not occur and after the reaction time neither the acetal product or silyl ether intermediate could be isolated. NMR and GCMS studies showed that the TBAF reaction had caused decomposition of the silyl ether intermediate but the decomposition products could not be isolated or identified.

The successfully synthesised silyl acetal was subjected to the P-K reaction conditions. Again the cyclisation was not successful and all that was recovered from the reaction was the un-reacted starting material. Following the difficulties with the synthesis of the silyl acetals and the failure of the silyl acetals to undergo cyclisation it was decided to stop the research into these temporary tethers.

### iii) Silicon as the tethering atom

**Scheme 18:**
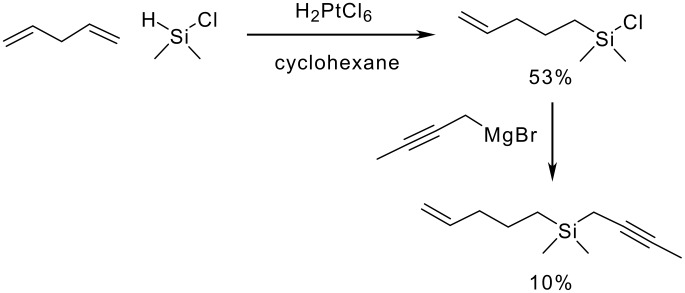
Preparation of silicon-tethered Pauson-Khand precursors.

It was decided that the final silicon tethering species that should be investigated was analogous to the first intramolecular P-K cyclisation demonstrated by Schore and co-workers.[34] The idea was to replace directly the carbon atom with a Si(Me)_2_ unit. This could provide results on both whether the P-K cyclisation of these species could be achieved and whether the Si-C bond was more stable towards the reaction conditions. There are three primary methods for the synthesis of silicon-carbon bonds: lithium-halogen exchange, Grignard-based methodologies and catalytic hydrosilylation. The approach decided upon used the Grignard and hydrosilylation reactions. Work by Swisher and Chen showed that by using a solution of chloroplatinic acid (H_2_PtCl_6_ in isopropyl alcohol) compounds containing terminal double bonds could be added catalytically to silane species to yield the substituted chlorosilanes.[33–34] This methodology was coupled with a Grignard reaction to attempt to cyclise the substituted silane (Scheme 18).

The catalytic hydrosilylation using chloroplatinic acid as the catalyst proved to be successful yielding the desired chlorosilane with a yield of 53% which is significantly more than that stated by Swisher and Chen for the same compound. However the Grignard reaction could only be accomplished in very low yield (10%). Nevertheless, the material obtained was subjected to the P-K reaction conditions, and, as before, failed to give any of the 5,7 tethered adduct. Starting material and some decomposed material was recovered.

**Scheme 19:**
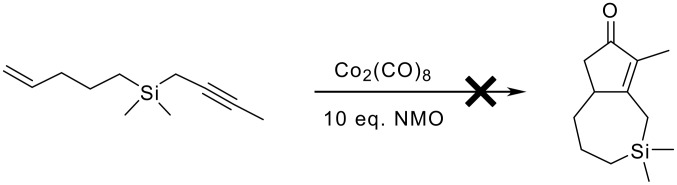
Failed Pauson-Khand reaction of a silicon-tethered substrate.

## Conclusion

In conclusion it can easily be seen from the results that silyl ethers and silyl acetals are not good substrates for the P-K reaction when using the standard stoichiometric NMO promoted conditions. Only di*iso*propylsilanes based silyl ethers have shown any potential as a tethered substrate for the reaction. However, further work is required to optimise the reactions using the di*iso*propylsilyl tethers and to develop an efficient route for their cleavage.

## Supporting Information

File 1 Representative Experimental Procedures and Characterisation Data for Si-tethered P-K reactions.
