# Supplementary material for: The use of silicon-based tethers for the Pauson-Khand reaction
Source: Beilstein J Org Chem. 2007 Jul 6;3:21. doi: 10.1186/1860-5397-3-21 (PMC1949821; doi:10.1186/1860-5397-3-21)

**The use of silicon-based tethers for the Pauson-Khand reaction**

Adrian P. Dobbs,*^a,b^ Ian J. Miller^b^ and Saša Martinović^b^

^a^ School of Biological and Chemical Sciences, Queen Mary University of London, Mile End Road, London E1 4NS, UK.

^b^ Department of Chemistry, University of Exeter, Stocker Road, Exeter, E4 4QD, UK.

Authors' Email Addresses:

A.Dobbs@qmul.ac.uk; I.J.Miller@exeter.ac.uk; martinovicsasa2002@yahoo.co.uk

**Representative experimental details:**

Petroleum ether or petrol refers to the fraction boiling between 40 °C and 60 °C. Dichloromethane and xylene were distilled over calcium hydride. Diethyl ether, THF and toluene were distilled over sodium and benzophenone, which was used as an indicator. All other solvents were obtained anhydrous form Aldrich and used directly into the reaction vessel. All reactions were carried out under an atmosphere of nitrogen unless otherwise stated using vacuum/nitrogen manifold. All glassware, syringes and needles were pre-dried in an oven (120-140° C) and cooled in a nitrogen atmosphere prior to use. Stirring was by internal magnetic follower. All chemicals were purified by distillation or re-crystallation where appropriate. Commercially available compounds were generally used without further purification. All reactions were followed by TLC. Analytical thin layer chromatography was carried out using aluminium backed plates coated with Merck Kieselgel 60 GF_254_. Plates were visualized under UV light (at 254 nm) or by staining with acidic potassium permanganate followed by heating. Flash chromatography was carried out using Matrex silica 60, 230-400 mesh; samples were applied as a saturated solution in an appropriate solvent. Infrared (IR) spectra were recorded in the range 4000-600 cm^-1^ using a Nicolet Magna IR 550 spectrometer with internal calibration. Spectra were recorded as KBr discs or as thin films between NaCl plates. Proton (^1^H) NMR spectra at 300 MHz and carbon (^13^C) NMR spectra at 75MHz were recorded on a Bruker AV 300 instrument in deuterated solvents and at 400 MHz and 100 MHz on an Avance DRX 400 instrument in deuterated solvents. NMR chemical shifts (δ) are quoted in ppm (parts per million) relative to an internal standard (CDCl_3_). High and low resolution mass spectra were recorded on a ThermoQuest Trace GC 2000 series or an Agilent 6890 Series GC System, Micromas GCT. Melting points were determined using a Gallenkamp melting point apparatus.

**Allyldi*iso*propylprop-2-ynyloxysilane**

To dry THF (25 ml) was added dichlorodi*iso*propylsilane (925 mg, 5 mmol) and the resulting solution was cooled to 0 °C with stirring. To this was added allylmagnesium chloride (2.50 ml of 2M solution in THF, 5 mmol) drop wise over 30 mins, maintaining the temperature at 0 °C. The solution was allowed to stir at this temperature for 3 hrs, after which triethylamine (1.01 g, 1.39 ml, 10 mmol), DMAP (61 mg, 0.5mmol) and propargyl alcohol (280 mg, 0.30 ml, 5 mmol) in dry THF (7 ml) were added and the resulting reaction allowed to warm to room temperature overnight. After this time the reaction was quenched with water (35 ml) and diluted with diethyl ether (35 ml). The organic phase was separated and the remaining aqueous phase extracted with diethyl ether (3 x 40 ml). The organic phases were combined, dried over anhydrous magnesium sulfate and concentrated *in vacuo*. The oily residue was purified by flash chromatography on neutral alumina gel eluting with 20:1 petroleum ether/diethyl ether to yield the *title compound* (430 mg, 41%) as a colourless oil. R_f_ = 0.25 (20:1 petroleum ether/diethyl). ν_max_ (neat) 3311 (Alkyne CH stretch), 2894 (CH_2_, CH_3_), 2122 (alkyne CC stretch), 1630 (non-conjugated double bond), 1090 (Si-O stretch); δ_H_ (300 MHz, CDCl_3_) 5.82 (1H, m, CH_2_=C**H**), 4.95-4.76 (2H, m, C**H**_2_=CH), 4.31 (2H, d, *J*= 2.4, O-C**H**_2_), 2.34 (1H, t, *J*= 2.4, C≡C**H**), 1.69 (2H, d, *J*= 8.0, Si-C**H**_2_), 1.00 (14H, s, 2 x C**H**_3_-C**H**-C**H**_3_), δ_C_ (75 MHz, CDCl_3_) 134.6 (CH_2_=**C**H), 114.4 (**C**H_2_=CH), 82.6 (C≡**C**H), 73.3 (**C**≡CH), 52.2 (O-**C**H_2_), 19.1 (Si-**C**H_2_), 17.8, 17.7 (**C**H_3_), 12.8 (2 x **C**H); *m/z* 211.2 (M+H, 1.7%), 171.1 (M-C_3_H_3_, 7.3%), 167.1 (M-C_3_H_5_, 100%); Found 211.1523, C_12_H_23_OSi (M+H) requires 211.1518; Anal. Calcd for C_12_H_22_OSi: C, 68.5, H, 10.5. Found C, 68.9, H, 10.2.

**3,3-Di*iso*propyl-3-sila-4-oxabicyclo[4.3.0]non-6-en-8-one**

To a stirred solution of allyldi*iso*propylprop-2-ynyloxysilane (75 mg, 0.36 mmol) in dry dichloromethane (10 ml) was added dicobalt octacarbonyl (134 mg, 0.39 mmol) and left to stir for 30 mins. After this period *N*-methylmorpholine *N*-oxide (487 mg, 3.6 mmol) was added batch wise to the reaction. The reaction was left to stir at room temperature for 72 hours. The resulting blue reaction was filtered through a layered celite/silica gel plug eluting with dichloromethane. The filtrate was collected and washed with water (15 ml) before being dried over anhydrous magnesium sulfate and being filtered. The filtrate was concentrated *in vacuo*. The oily residue was purified by flash chromatography on silica gel eluting with 2:1 dichloromethane/ethyl acetate to yield the *title compound* (**40**) (64 mg, 75%) as a yellow oil. R_f_ = 0.57 (2:1 dichloromethane/ethyl acetate). ν_max_ (neat) 2946(CH_2_), 1713 (CO), 1686 (conjugated alkene), 1265 (Si-*i*Pr), 1086 (Si-O); δ_H_ (300 MHz, CDCl_3_) 5.77 (1H, s, C**H**=C), 4.78 (1H, d, *J*= 17.4, O-C**H**_2_), 4.67 (1H, d, *J*= 17.4, O-C**H**_2_), 3.19-3.14 (1H, m, CH_2_-C**H**-CH_2_), 2.65 (1H, dd, *J*= 6.2, 18.8, CH-C**H**_2_-CO), 1.97 (1H, dd, *J*= 1.7, 18.8, C**H**_2_-CO), 1.15 (1H, dd, *J=*5.5, 14.5, Si-C**H**_2_), 0.97 (7H, s, *i*-Pr), 0.93 (7H, s, *i*-Pr), 0.62 (1H, collapsed dd, Si-C**H**_2_); δ_C_ (75 MHz, CDCl_3_) 208.1 (**C**O), 181.4 (CH=**C**), 128.2 (**C**H=C), 64.3 (O-**C**H_2_), 46.4 (CO-**C**H_2_), 37.4 (**C**H), 17.3 (4 x **C**H_3_), 14.9 (Si-**C**H_2_), 12.6 (2 x **C**H); *m/z* 239.1 (M+H, 100%), 195.1 (M-*i*-Pr, 11.3%); Found 239.1485, C_13_H_22_OSi (M+H) requires 239.1467; Anal. Calcd for C_13_H_22_O_2_Si: C, 65.5, H, 9.30. Found C, 65.1, H, 9.64.

**3,3-Di*iso*propyl-3-sila-4-oxabicyclo[4.3.0]non-6-en-8-one**

**THF-insertion adduct obtained from the reaction with tungsten pentacarbonyl**

(possible structure)

A stirred solution of tungsten hexacarbonyl (425 mg, 1.2 mmol) in dry THF (20 ml) was irradiated in a rayonet for 1.5 h until the solid tungsten hexacarbonyl was no longer visible and the solution had turned a green/yellow colour. After this period allyldimethylpent-4-ynyloxy silane (200 mg, 1.1 mmol) was added drop wise to the solution and the resulting mixture was heated at 83˚ C for 48 hrs. After this period carbon monoxide was slowly bubbled through the mixture for 2 hrs. The mixture was filtered through a layered celite and silica gel plug eluting with dichloromethane. The filtrate was washed with water (20 ml) and the aqueous phase was extracted with diethyl ether (3 x 15 ml). The organic phases over combined, dried with anhydrous magnesium sulfate, filtered and concentrated *in vacuo*. The oily residue was purified by flash chromatography on silica gel eluting with 9:1 petrol/ethyl acetate to yield the *title compound* (**31**) (79 mg, 47%) as a colourless oil. R_f_ = 0.65 (9:1 Petrol/Ethyl Acetate). ν_max_ (neat) 3431 (OH), 2986 (CH_2_), 1655 (alkene), 1096 (cyclic ether); δ_H_ (300 MHz, CDCl_3_) 5.12 (1H, dd, *J*= 0.88, 1.46, C**H**=C), 3.90-3.85 (2H, m, C**H**_2_-O), 3.76-3.70 (1H, m, C**H**_2_-C_(quat)_), 3.50-3.45 (1H, m, C**H**_2_-C_(quat)_), 2.27-2.24 (2H, m, C**H**_2_), 1.95-1.59 (7H, m, C**H** + 2(C**H**_2_)); δ_C_ (75 MHz, CDCl_3_) 103.8 (**C**H), 83.9 (**C**_(quat)_), 68.4 (**C**_(quat)_), 68.8 (**C**H_2_), 65.4 (**C**H_2_), 32.3 (**C**H_2_), 28.6 (**C**H_2_), 15.3 (**C**H_2_); *m/z* (CI) 154.2 (M^+^, 1.1%), 153.2 (M-H, 6.1%), 111.1 (M-C_2_H_5_O, 14.3%); Found 155.1060, C_9_H_15_O_2_ (M+H) requires 155.1072.


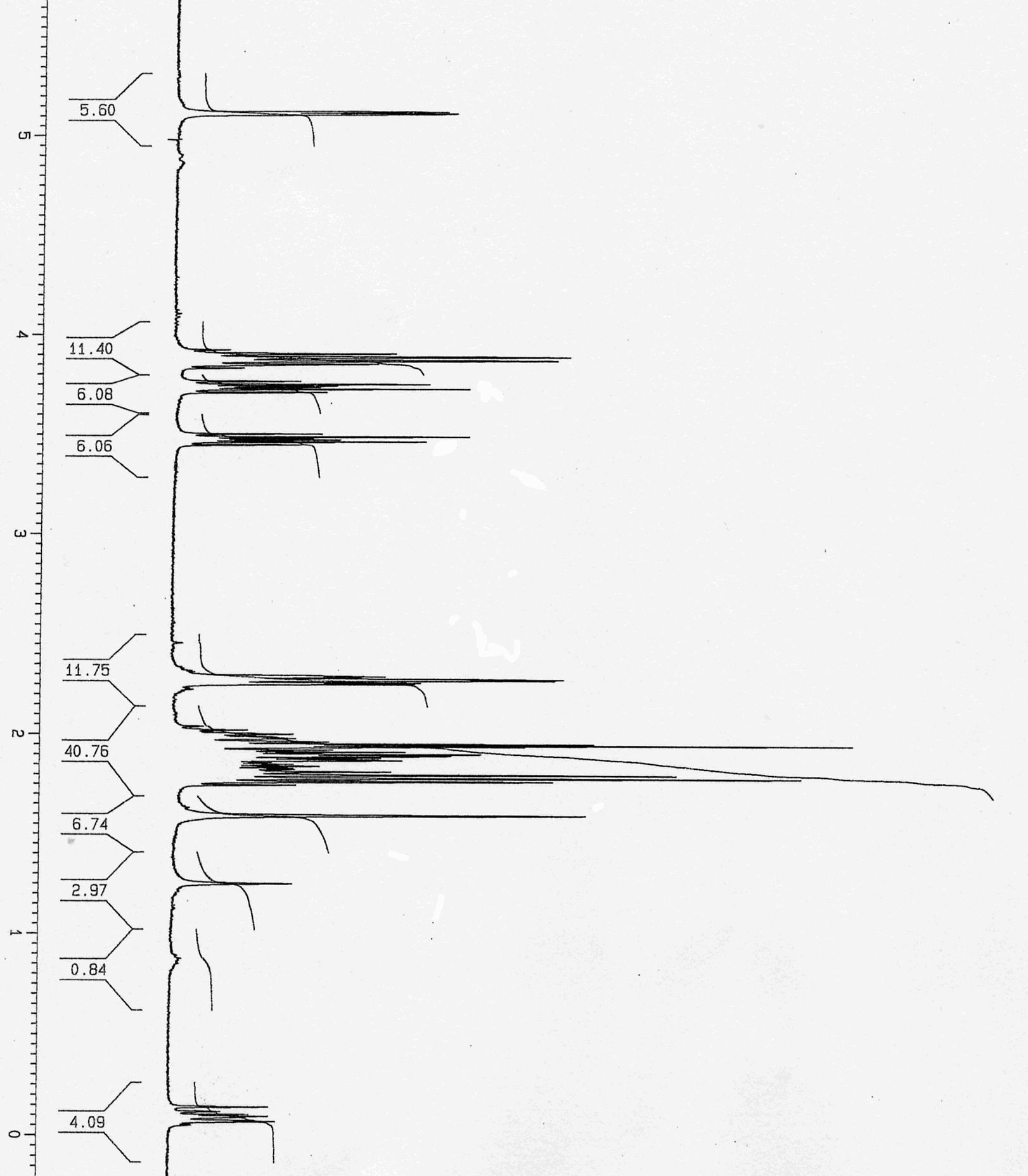


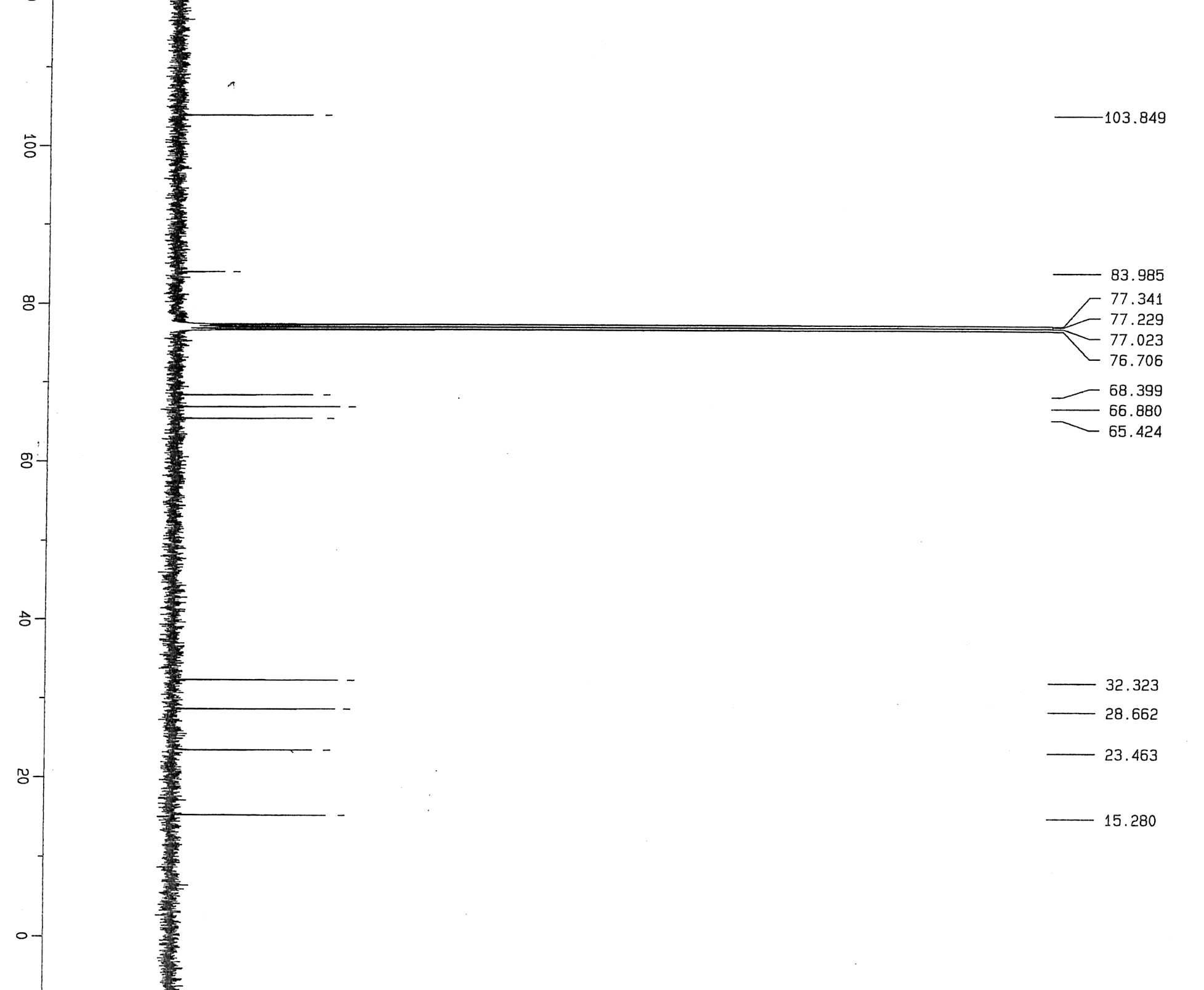

Supplement: File 1 — Representative Experimental Procedures and Characterisation Data for Si-tethered P-K reactions. [file Beilstein_J_Org_Chem-03-21-s001.doc]
